# Climate Crisis in the Mediterranean Hotspot: Conservation Challenges for Endangered Salamanders in Southern Türkiye

**DOI:** 10.1002/ece3.73962

**Published:** 2026-07-19

**Authors:** Morteza Naderi, Kerim Çiçek, Yasin İlemin, Amaël Borzée, Hossein Azadi, Babak Naimi

**Affiliations:** ^1^ Department of Biology, Faculty of Sciences Sakarya University Sakarya Türkiye; ^2^ Department of Biology, Faculty of Sciences Ege University Izmir Türkiye; ^3^ Department of Environmental Protection and Technologies, Fethiye A.S.M.K. Vocational School Muğla Sıtkı Koçman University Muğla Türkiye; ^4^ Lab of Animal Behaviour and Conservation, College of Life Sciences Nanjing Forestry University Nanjing China; ^5^ Amphibian Specialist Group, Species Survival Commission International Union for the Conservation of Nature Gland Switzerland; ^6^ State Key Laboratory of Ecological Safety and Sustainable Development in Arid Lands, Xinjiang Institute of Ecology and Geography Chinese Academy of Sciences Urumqi China; ^7^ Department of Geography Ghent University Ghent Belgium; ^8^ Quantitative Biodiversity Dynamics (QBD), Department of Biology University of Utrecht Utrecht the Netherlands

**Keywords:** CMIP6/SSP, ensemble species distribution models, *Lyciasalamandra*, Mediterranean hotspot, microrefugia, Türkiye

## Abstract

Amphibians are particularly vulnerable to climate change, with many species projected to experience significant reductions in their current distributional ranges due to ongoing climatic impacts. Mediterranean biodiversity hotspots, characterised by exceptionally high species richness, including rare and endemic species, are especially susceptible. Here, we employed an ensemble of eight advanced distribution modelling algorithms to model and explain the spatial distribution of the seven closely related salamander species and one documented hybrid lineage, all belonging to the genus *Lyciasalamandra*, within a Mediterranean hotspot in Türkiye. We explored how their spatial distributions are linked to both climate and anthropogenic activities and examined the projected changes in suitable habitat. Projected habitat suitability maps, under various climate change scenarios for the coming decades, indicate a severe decline in the quality and extent of habitats for Lycian salamanders, raising concerns about their long‐term survival. Overall, our findings emphasise the urgency of species‐specific conservation actions that prioritise the protection of microrefugia and the maintenance of ecological connectivity to enhance the long‐term persistence of Lycian salamanders under accelerating climate change.

## Graphical abstract

Our ensemble modelling shows that all Lyciasalamandra species face pronounced declines in suitable habitat under future climate scenarios. Climate and human pressures jointly drive these contractions, threatening the long‐term persistence of several species. These findings highlight the urgent need for microrefugia protection and enhanced ecological connectivity in Mediterranean biodiversity hotspots.

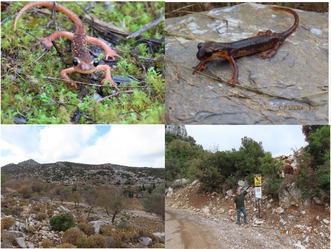

## 1 Introduction

Climate change is a major driver of accelerating biodiversity loss worldwide, and amphibians remain the planet's most threatened vertebrate group (Luedtke et al. 2023). For many amphibian species, rapid warming, habitat loss, and increasing human pressures are leading to range shifts, population declines, and sharply elevated extinction risk (IUCN SSC Amphibian Specialist Group 2024), and climate change is having increasingly negative impacts (Pottier et al. 2025). Salamanders are especially vulnerable due to their permeable skin, limited dispersal ability, and dependence on cool, humid microhabitats in both terrestrial and aquatic systems (Wake and Vredenburg 2008), with more than 60% of species threatened with extinction (Luedtke et al. 2023).

Mediterranean ecosystems, renowned for high biodiversity and endemism, now face intensifying climate threats (Aurelle et al. 2022; Hingnekar and Dhadse 2025). Warming, drying, and extreme events—such as prolonged drought, heatwaves, and wildfires—are projected to increase even under low‐warming futures, threatening native flora and fauna and eroding ecosystem integrity (Lindner et al. 2008). Within amphibians, salamanders are recognised for their acute sensitivity to environmental change (Ashrafzadeh et al. 2019), underscoring their priority for conservation action.

In the Mediterranean Basin, shifts in fire regimes have emerged as a key manifestation of the climate crisis, with hotter, longer fire seasons and an increasing frequency of extreme “megafires” documented across southern Europe (Turco et al. 2017; Moreira et al. 2020; Bowman et al. 2009). Historically, wildfires functioned as natural, moderate disturbances that maintained structural heterogeneity and nutrient cycling in *Pinus brutia* and other Mediterranean forest types, but climate warming, land‐use change, and fire suppression have jointly driven a transition towards larger, more severe fires with long‐lasting ecological impacts (Pausas and Keeley 2019; Keeley et al. 2020). Lycian salamanders (*Lyciasalamandra* spp.) offer a classic example of micro‐endemism and climate sensitivity in the Mediterranean. This genus comprises seven allopatric, viviparous species, including *L. antalyana* , *L. atifi* , *L. billae* , *L. fazilae* , *L. flavimembris* , *L. helverseni* , and *L. luschani* , each restricted to fragmented karstic limestone habitats in southwestern Türkiye and adjacent Aegean islands (Sparreboom 2014; Veith et al. 2016, 2020; Dinis et al. 2025). Their life history traits, including terrestrial reproduction and subterranean refuge use, confer high susceptibility to warming and drying, while highly fragmented ranges further increase vulnerability.

In southwestern Anatolia, these salamanders are closely associated with Turkish red pine ( *Pinus brutia* ) forests and surrounding maquis and rocky habitats, where canopy cover, coarse woody debris, and boulder fields generate the cool, humid microsites required for activity and estivation (Boydak 2004; Barbati et al. 2013; Gülçin et al. 2022; Yousefkhani et al. 2023). Recent mega‐fires in such forests, including the 2021 events in southwestern Türkiye, have already been shown to damage or eliminate key microrefugia for *Lyciasalamandra* populations, with severe implications for local persistence (Gülçin et al. 2022).

Despite increasing attention to Mediterranean amphibian conservation, species‐level forecasts that integrate climate and human disturbance, and explicitly identify climate‐change microrefugia, remain rare for *Lyciasalamandra*. Microrefugia, small landscape features buffered against regional climate change through topography, cold‐air pooling, or local moisture retention, may be critical for persistence when broader climatic suitability declines. Moreover, the interaction between climate‐driven habitat contraction and post‐fire forestry practices has received little attention, even though management decisions taken immediately after wildfires can either preserve or destroy the last remaining moist refuges for these salamanders (Certini 2005; Lindenmayer and Franklin 2002; Lindenmayer et al. 2000). All target species represent ecologically and evolutionarily distinct populations of high conservation priority, making them ideal subjects for integrated multi‐species SDM analysis.

This study employs ensemble species distribution modelling for all target *Lyciasalamandra* species to (i) quantify present habitat suitability relative to climate and human impacts, (ii) project changes under multiple future CMIP6/SSP scenarios, and (iii) pinpoint persistent microrefugia and key habitat corridors that could sustain species under accelerating change. By linking distributional shifts, refugial persistence, and ecological connectivity, we provide decision support for conservation planning in this rapidly warming Mediterranean hotspot.

## 2 Materials and Methods

### 2.1 Study Area

This study focuses on Mediterranean biodiversity hotspots in southwestern Türkiye, a region within the Mediterranean Basin recognised for exceptional species richness, high endemism, and mounting threats from climate change and human activity (Médail and Myers 2004). The landscape encompasses a mosaic of ecosystems, coastal habitats, maquis shrublands, diverse forest types, and montane zones throughout the Taurus Mountains, each supporting rich and specialised plant and animal communities. Coastal areas host wetlands, rocky shores, and beaches vital for numerous species, while evergreen maquis shrublands boast a high diversity of endemic flora. Forests dominated by *Pinus brutia* and oak (*Quercus* spp.), mixed with conifers and broadleaf species, contribute to ecological complexity and play key roles in water regulation, soil protection, and habitat provision. The elevation gradients in the Taurus Mountains additionally create alpine and subalpine refugia for climate‐sensitive taxa.

Southwestern Türkiye experiences a hot, dry Mediterranean climate (Köppen Csa; Beck et al. 2018). Lycian salamander habitats are concentrated in Muğla and Antalya provinces. In Muğla, air temperatures range from 9.9°C to 22.0°C, with annual averages of 15.4°C and 1059 mm precipitation. Antalya is warmer, with temperatures from 14.1°C to 24.7°C and similar annual rainfall. Key localities such as Marmaris National Park, Göcek‐Köyceğiz Specially Protected Area, Köprülü Canyon National Park, and Mount Sandras are notable for their biodiversity and conservation importance.

### 2.2 Target Species

**FIGURE 1:**
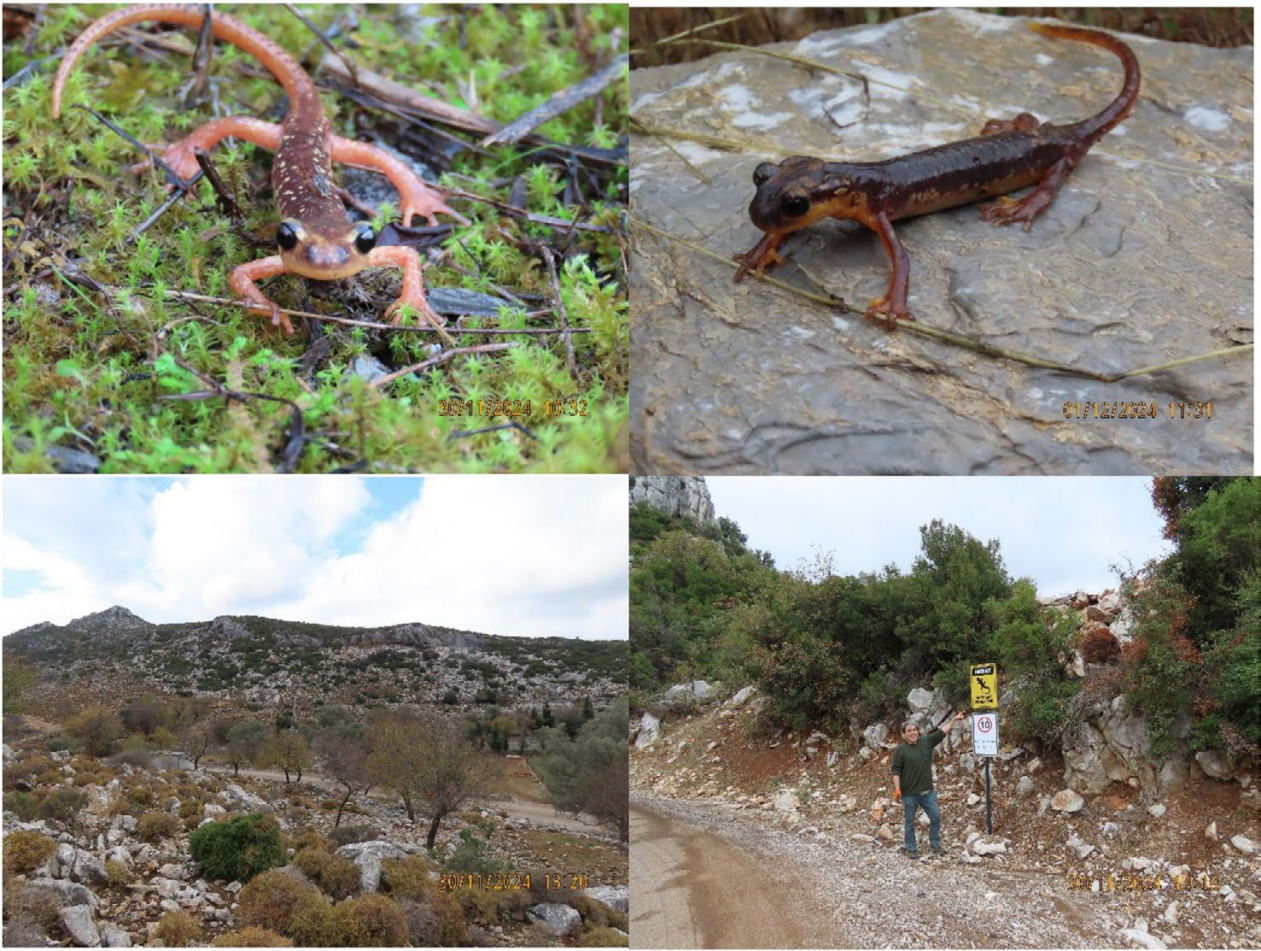
Representative photographs of two *Lyciasalamandra* species and their associated microhabitats in the Muğla region, southwestern Türkiye (December 2024). Top row: *L. flavimembris* —(left) individual in situ on karstic limestone substrate; (right) typical *Pinus brutia* forest habitat with coarse woody debris and boulder field. Bottom row: *L. fazilae* —(left) individual sheltering beneath rock overhang; (right) north‐facing maquis‐pine ecotone showing characteristic estivation microhabitat. All photographs by the first author.

Lycian salamanders (*Lyciasalamandra* spp.) are slender, viviparous, and terrestrial amphibians distinguished by their adaptation to a range of Mediterranean habitats. They occupy elevations from near sea level up to 1340 m, most commonly on north‐facing slopes, pine forests, and maquis shrublands (Veith et al. 2001; Sparreboom 2014; Rödder et al. 2011; Sinsch et al. 2017; Yaşar et al. 2021). These salamanders are particularly well‐adapted to the region's extremes, sheltering deep within humid crevices of boulder fields and karstic limestone slopes—microhabitats that buffer against summer heat and winter dampness. Within their naturally restricted ranges, Lycian salamanders face persistent threats, including habitat loss from wildfires, fragmentation, and overcollection for scientific purposes. Their habitats in southwestern Türkiye span the provinces of Muğla and Antalya, while *L. helverseni* is endemic to Greece, confined to Karpathos, Kasos, and Saria in the southeastern Aegean Sea. Figure 1 provides representative views of two species ( *L. flavimembris* and *L. fazilae* ) alongside key habitat features.

### 2.3 Occurrence Data

Occurrence records for all *Lyciasalamandra* species were compiled from multiple sources: (1) systematic field surveys conducted by the authors between 2019 and 2025 using standardised nocturnal and diurnal transect walks in known habitat types (karstic limestone slopes, *Pinus brutia* forests, and maquis shrublands); (2) georeferenced records from published herpetological surveys and peer‐reviewed literature (Arslan et al. 2018, 2020; Yousefkhani et al. 2023); and (3) verified records shared by regional herpetologists and museum collections. All records were georeferenced to WGS84 and assigned a collection date. A spatial thinning procedure was employed to sample from nearby records within 2.5 arc‐minute geographic distance, which can reduce the risk of spatial autocorrelation. After cleaning, the final dataset comprised 424 records distributed across all species (per‐species counts are provided in Supplementary Table S1).

In addition to the seven recognised species, we included *L. antalyana ×billae*, a documented hybrid lineage arising from the contact zone between *L. antalyana* and *L. billae* in southwestern Türkiye (Veith et al. 2016; Scott et al. 2025). This hybrid taxon was treated as a separate analytical unit, given its distinct geographic occurrence and ecological characteristics. In accordance with Ecology and Evolution exceptions for threatened taxa, spatial coordinates for all *Lyciasalamandra* species, which are legally protected and at documented risk of illegal collection for the pet trade, are provided at a coarsened resolution of 10 km to mitigate poaching risk. Full‐resolution locality data are available to researchers upon reasonable request to the corresponding author, subject to a data‐sharing agreement. All other biological parameters, environmental covariates, and modelling outputs remain at the resolution used for the analyses to ensure full study replicability.

### 2.4 Predictor Preparation

Nineteen bioclimatic variables representing temperature and precipitation patterns were obtained from WorldClim version 2.1 at a spatial resolution of 2.5 arc‐minutes (approximately 4.6 km at the equator; Fick and Hijmans 2017). These variables were selected for their biological relevance to amphibian ecology and their capacity to capture key aspects of climatic variability. Topographic variables, elevation, slope, aspect, and terrain ruggedness index (TRI) were derived from the Shuttle Radar Topography Mission (SRTM) digital elevation model and projected to Lambert Azimuthal Equal‐Area (ETRS 1989 LAEA) before being reprojected to WGS84 for consistency with other layers (Sillero and Barbosa 2021). To reduce multicollinearity among bioclimatic predictors, a Variance Inflation Factor (VIF) analysis was performed using the usdm R package (Naimi 2023), iteratively removing variables with VIF > 10 until all retained variables had acceptable collinearity levels (Naimi et al. 2014). Anthropogenic factors were also included as indirect predictors, variables that do not directly control species physiology but shape habitat availability and accessibility through their influence on land cover, disturbance regimes, and resource availability. These included distances to farmland, forests, roads, villages, and water resources, as well as population density and the Human Footprint Index (HFI). Forest and farmland layers were extracted from the Corine Land Use Land Cover 2018 Vector Dataset. Road network data were retrieved from OpenStreetMap, with attributes including motorways, trunk roads, primary roads, secondary roads, and their linked roads. Proximity to these variables was calculated using the Euclidean Distance tool in ArcGIS Pro 2.8. Population density and the Human Footprint Index (HFI) database (Venter et al. 2016) were obtained from the Socioeconomic Data and Application Center (SEDAC) database.

### 2.5 Model Fitting

To forecast salamander distributions under present and future climate conditions, we constructed habitat models using an ensemble framework within the R environment, primarily leveraging the sdm package (Naimi and Araújo 2016; Naimi et al. 2026). We followed the methodology of Ebrahimi et al. (2025), applying eight widely used statistical and algorithmic approaches that provide both parametric and non‐parametric methods for species distribution modelling: Generalised Linear Models (GLM; McCullagh and Nelder 1989), Boosted Regression Trees (BRT; Elith et al. 2008), Multiple Adaptive Regression Splines (MARS; Friedman 1991), Random Forests (RF; Breiman 2001), Support Vector Machines (SVM; Cortes and Vapnik 1995), Multi‐layer Perceptrons (MLP; Haykin 1994), Maximum Entropy (Maxent; Phillips et al. 2006), and Domain (Carpenter et al. 1993). Model calibration included generating 1000 randomly distributed background points from the study area to contrast with observed presences. For evaluation, we used the Area Under the Receiver Operating Characteristic Curve (AUC) and the True Skill Statistic (TSS), established metrics for model discrimination and accuracy (Allouche et al. 2006; Rausell‐Moreno et al. 2025).

Species records were partitioned into training and testing sets using a repeated bootstrapping strategy, generating 10 replications for each species. The bootstrapping approach uses a sampling with replacement method to draw species records of the same size as the original records, which were used as the training dataset. The records that were not selected for training were then identified and used as the testing dataset. Each data replicate was used across all eight modelling algorithms, resulting in 80 models per species. To reduce the risk of spatial autocorrelation resulting from spatially clustered records, a spatial thinning procedure was employed before modelling by sampling species records within a geographic distance of 2.5 arc‐minutes. Background points (*n* = 1000) were drawn randomly from the full extent of the study area, which encompasses the entire known range of all species plus a buffer zone. This background selection strategy reflects the accessible area available to the species and is appropriate for presence‐background modelling frameworks (Phillips et al. 2006; Naimi and Araújo 2016). To control for overfitting, only models with TSS > 0.6 were retained for ensemble averaging, and predictions were weighted by individual AUC scores to down‐weight poorly performing models (Araújo and New 2007; Naimi and Araújo 2016; Ebrahimi et al. 2023; Naimi et al. 2022). This approach provides a more uncertainty‐aware and reliable consensus prediction.

### 2.6 Model Evaluation

Model performance was assessed using two complementary metrics: the Area Under the Receiver Operating Characteristic Curve (AUC; Swets 1988) and the True Skill Statistic (TSS; Allouche et al. 2006). TSS accounts for both omission and commission errors and ranges from −1 (no better than random) to +1 (perfect agreement). Only models with TSS > 0.6 were retained for ensemble averaging, and ensemble predictions were weighted proportionally to individual model AUC scores (Araújo and New 2007). Species‐level AUC and TSS values, along with per‐algorithm performance, are reported in Table 2. Sensitivity and specificity at the TSS‐maximising threshold are also reported in Table 2 for each species. We note that random bootstrapping rather than spatial cross‐validation was used for model evaluation; spatial autocorrelation among occurrence records was not formally assessed, which we acknowledge as a limitation that may result in slightly optimistic performance estimates for species with spatially clustered records (Valavi et al. 2019).

### 2.7 Projection and Dispersal Scenarios

We used bioclimatic variables at a 2.5 arc‐minute resolution from the WorldClim 2.1 representing the historical baseline period (1970–2000; Fick and Hijmans 2017). For future scenarios, we incorporated climate projections from the CMIP6 (Coupled Model Intercomparison Project Phase 6) suite, as referenced in the IPCC's Sixth Assessment Report (AR6). These projections used three widely applied general circulation models (GCMs)—MIROC6, CNRM‐CM6‐1, and MPI‐ESM1‐2‐LR—selected for their proven performance in ecological studies across Europe (Amindin et al. 2024; Naimi et al. 2022).

We modelled habitat suitability across two Shared Socio‐economic Pathways (SSPs) to represent a range of plausible futures: SSP3‐7.0 (moderate) and SSP5‐8.5 (pessimistic/high emissions). Projections were generated for both mid‐century (2050: mean of 2041–2060) and late‐century (2070: mean of 2061–2080) periods.

To address uncertainty and capture the range of potential climate outcomes, we expanded analyses using available data from distinct GCMs and two SSPs (3–7.0, 5–8.5) for additional intervals (2050, 2070, 2090), ensuring robust scenario coverage. For future projections, we accounted for three dispersal scenarios as per Ebrahimi et al. (2025): (1) unlimited dispersal (species can occupy all accessible suitable habitat), (2) no dispersal (restricted to current distribution), and (3) limited dispersal based on an inferred average rate of 0.5 km/year for salamanders (Smith and Green 2005).

### 2.8 Thresholding and Microrefugia Identification

Continuous suitability outputs were converted to binary presence–absence maps using the threshold that maximised TSS(max[sensitivity + specificity]), facilitating quantification of suitable habitat area and its temporal changes. From these, we further identified climate microrefugia—areas predicted to remain suitable under all future scenarios—which are critical for conservation planning as they represent potential strongholds against regional climate shifts (Ashcroft 2010). These persistent areas are treated as conservation priority zones for long‐term population persistence.

### 2.9 Niche Overlap Analysis

Pairwise niche overlap among all species was quantified using complementary indices computed in the sdm package (Naimi and Araújo 2016): the Sørensen similarity coefficient (*D*), Index of Model Similarity (*I*_mod_), Index of Correlation (*I*_cor_), Overall Niche Overlap (*O*), Range Overlap (*R*), Bhattacharyya coefficient (BC), and Pearson/Spearman correlation coefficient (COR), following standard niche overlap protocols (Warren et al. 2008). Together, these metrics capture overlap in environmental space, predicted distribution, and probability density, providing a multi‐faceted assessment of ecological similarity and potential co‐occurrence among species. All statistical and geospatial analyses were conducted in R using the terra package (Hijmans 2023) for raster processing, sdm for modelling, and usdm (Naimi 2023) for variable selection.

### 2.10 Uncertainty Handling

Uncertainty in future projections was quantified by running models across different GCMs (MIROC6, CNRM‐CM6‐1, MPI‐ESM1‐2‐LR), SSPs (3–7.0, 5–8.5), and time periods (2050, 2070), yielding a large ensemble of projections per species. Spatial agreement among projections was used to identify areas of high confidence (suitable under all combinations) and low confidence (suitable under only some combinations). Microrefugia were defined conservatively as cells suitable across all scenario combinations to maximise conservation relevance. Ensemble spread across algorithms was also used as an additional indicator of local prediction uncertainty.

## 3 Results

**FIGURE 2:**
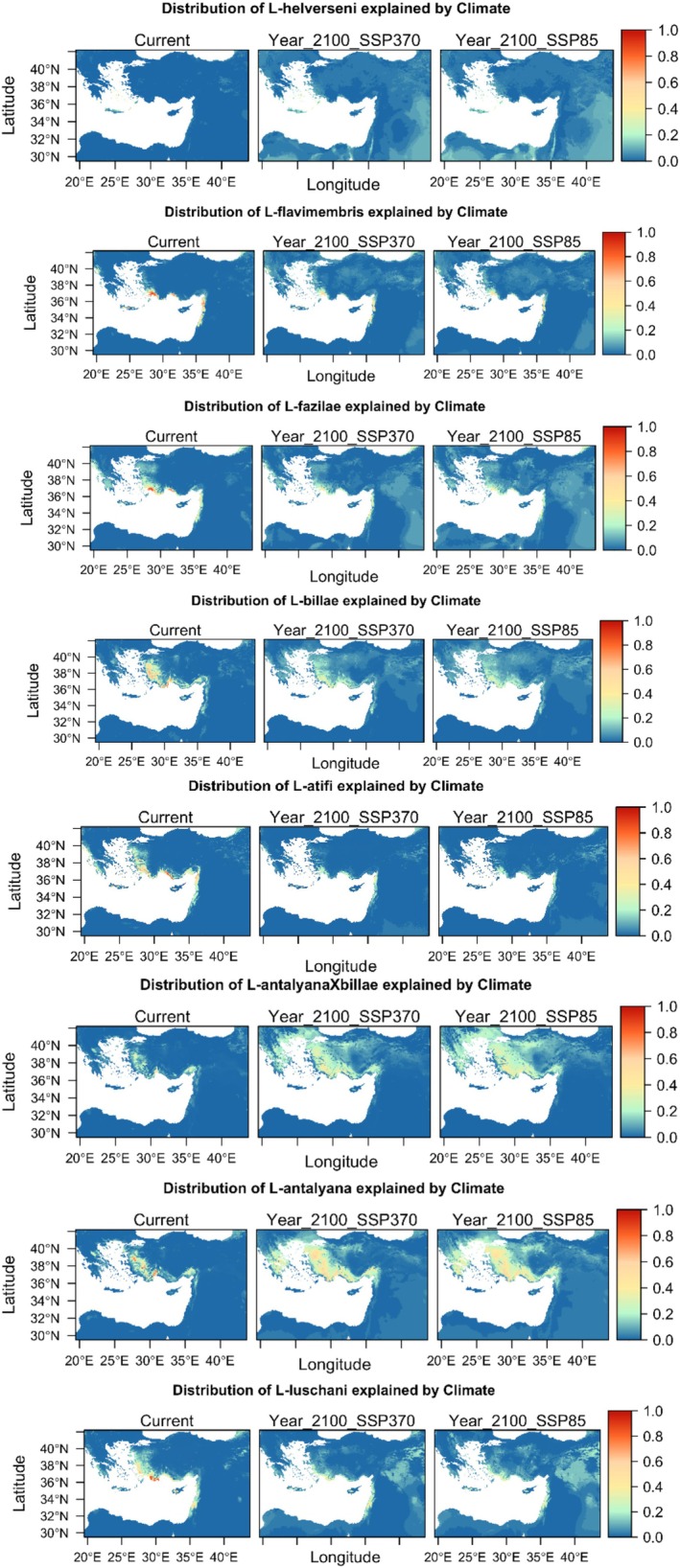
Distribution models of the target species only using climatic variables under different scenarios.

**FIGURE 3:**
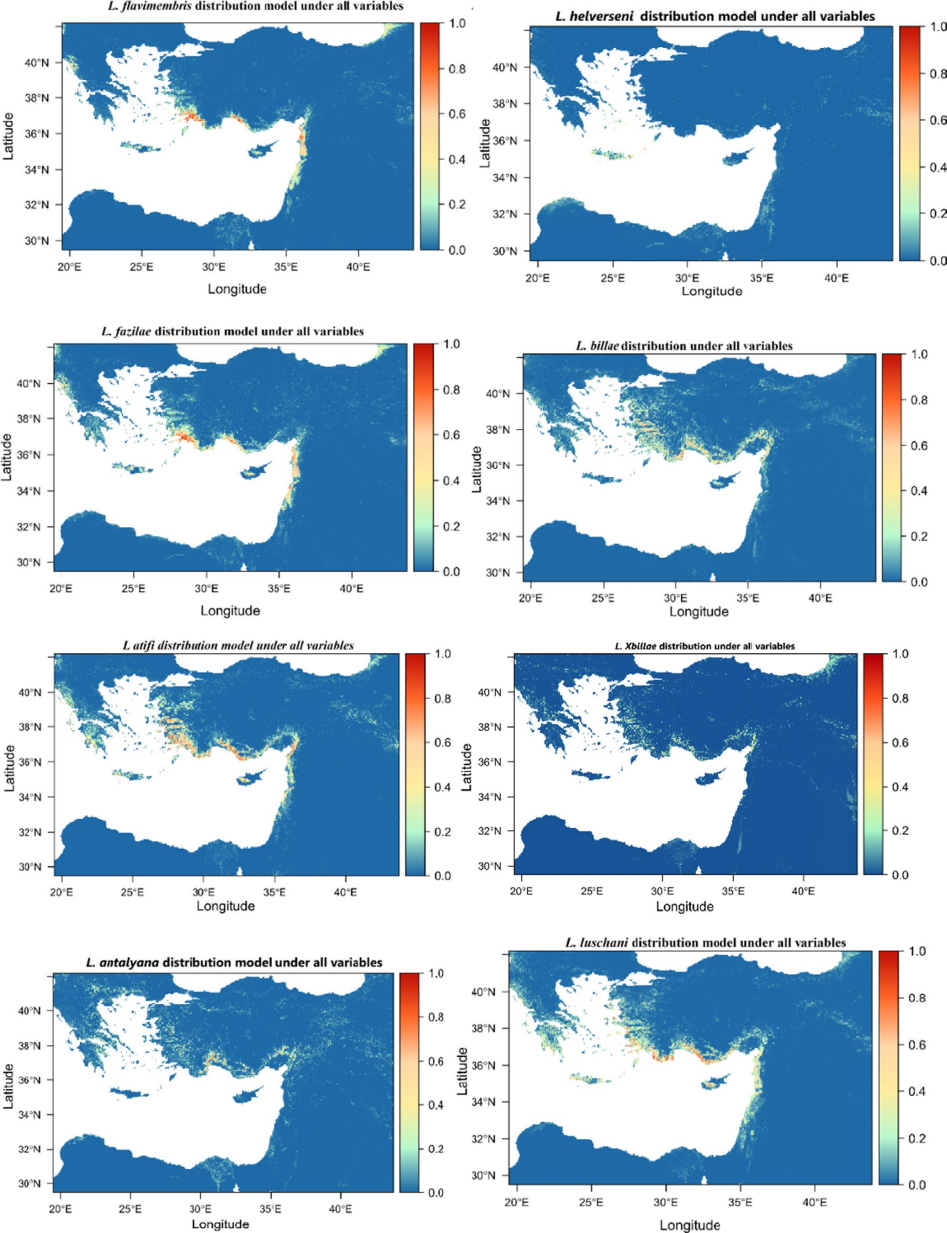
Species current distribution model using all variables, including climatic data.

**FIGURE 4:**
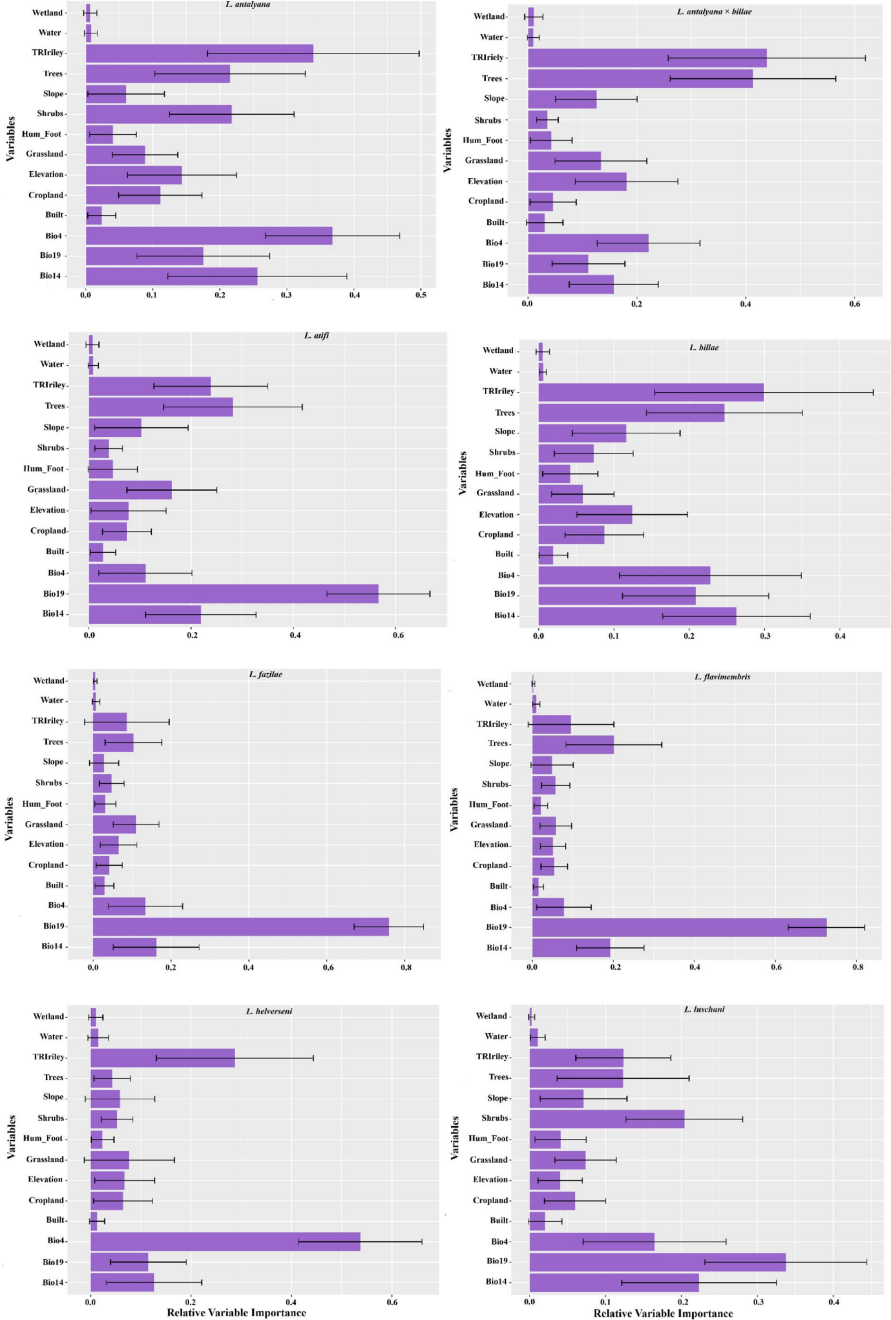
Variable importance scores for the most influential predictors included in the ensemble species distribution models for each of the *Lyciasalamandra* species. Each panel corresponds to one species (panels arranged as: *L. antalyana* , *L. antalyana* × *billae*, *L. atifi* , *L. billae* , *L. fazilae* , *L. flavimembris* , *L. helverseni* , and *L. luschani* ). Variables retained in the final models include bioclimatic variables (bio10: Mean temperature of the warmest quarter; bio13: Precipitation of the wettest month; bio14: Precipitation of the driest month; bio15: Precipitation seasonality; bio18: Precipitation of the warmest quarter; bio19: Precipitation of the coldest quarter), land cover variables (trees, shrubs, grasslands, croplands), topographic variables (slope, elevation), and the Human Footprint index (Hum_Foot). Higher values indicate a greater contribution of that variable to model performance. Variable importance was assessed across all algorithms using the weighted‐average ensemble approach.

**FIGURE 5:**
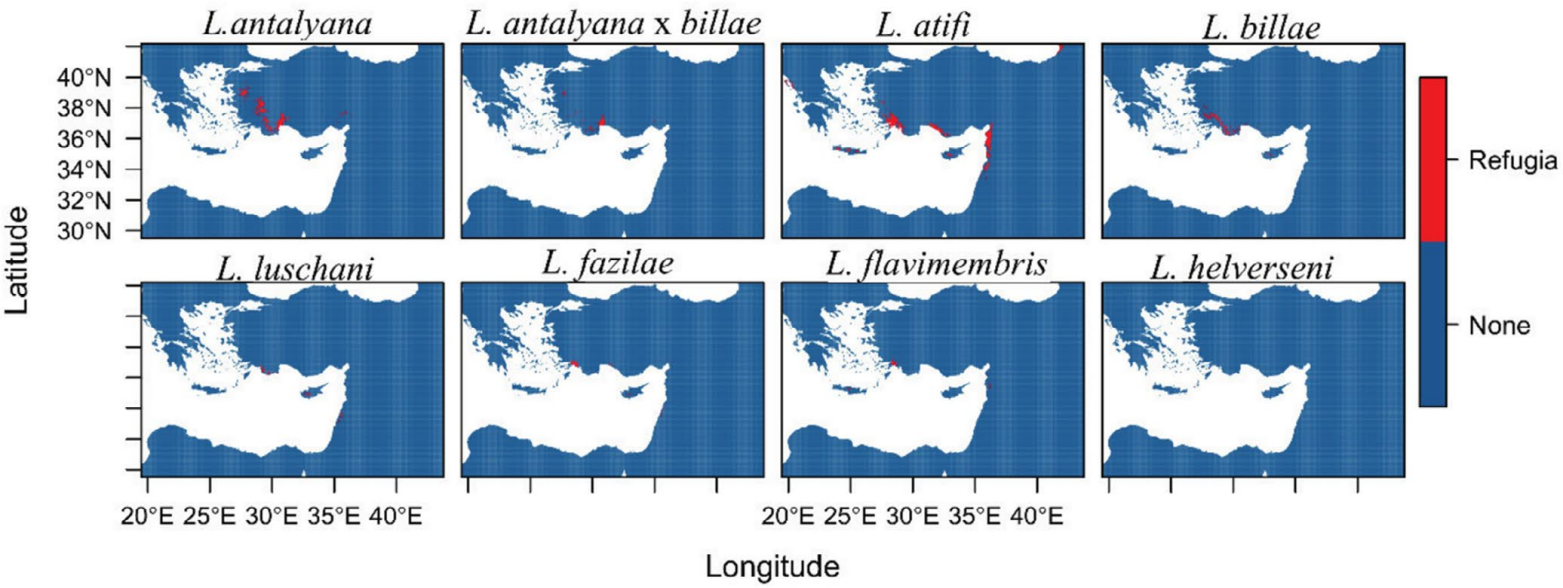
Geographic distribution of predicted climate microrefugia for all *Lyciasalamandra* species in southwestern Türkiye. Microrefugia are defined as grid cells (2.5 arc‐min resolution) projected to remain climatically suitable under all GCM–SSP combinations across all time periods (2050–2070). Each species is shown in a distinct colour. Grey shading = hillshade relief. Black outlines = existing protected area boundaries. The inset shows the location of the study area within Türkiye.

Upon collinearity screening of the bioclimatic predictors for species distribution models, 10 variables remained in the final models, all with variance inflation factor (VIF) values below 10: mean diurnal range (bio2), temperature annual range (bio7), mean temperature of wettest quarter (bio8), mean temperature of driest quarter (bio9), mean temperature of warmest quarter (bio10), precipitation of wettest month (bio13), precipitation of driest month (bio14), precipitation seasonality (bio15), precipitation of warmest quarter (bio18), and precipitation of coldest quarter (bio19) (Figures 2 and 3). When modelling with all bioclimatic and other biogeographic variables, these variables remained in the final models: trees, shrubs, grasslands, and croplands from plant‐related variables, slope and elevation from topographic variables, as well as human footprints (Figure 4). Model performance was evaluated using the True Skill Statistic (TSS) (Allouche et al. 2006), which considers both omission and commission errors and ranges from −1 (no better than random) to +1 (perfect agreement). An Area Under the Curve (AUC) score exceeding 0.9 indicated excellent predictive power (Swets 1988). To enhance reliability and reduce uncertainty, we retained only models with TSS scores greater than 0.6 and combined them using a weighted average (Araújo and New 2007; Thuiller et al. 2009; Naqibzadeh et al. 2022). The final ensemble model demonstrated robust predictive ability (Figure 2), with a mean AUC of 0.93 and a mean TSS of 0.81 across all species and scenarios. Among the suite of tested algorithms, BRT, MARS, SVM, MLP, and Domain exhibited the lowest AUC values (mean = 0.78), and SVM, MLP, and Domain exhibited the lowest TSS (mean = 0.65). In contrast, RF, MaxEnt, and GLM consistently achieved the highest AUC and TSS values (mean AUC = 0.95, mean TSS = 0.86), further supporting their value in robust ecological niche modelling. The ensemble habitat suitability maps (Figure 2) reveal that current climatically suitable areas for most species are concentrated in the rugged coastal and montane zones of Muğla and Antalya provinces, particularly on north‐facing slopes within *Pinus brutia* forest belts. The geographic distribution of predicted microrefugia, grid cells projected to remain suitable under all GCM–SSP combinations, is shown in Figure 5. Microrefugia are clustered primarily on north‐facing slopes within the Taurus Mountain foothills, with notable concentrations in the Göcek–Köyceğiz Specially Protected Area, Köprülü Canyon National Park, and the Mt. Sandras massif. Several microrefugia fall outside existing protected area boundaries, representing priority sites for future designation.

Under SSP3‐7.0 and especially SSP5‐8.5, suitable habitat contracts substantially and shifts upslope, with the most severe losses concentrated at lower elevations and southern aspects. *L. billae* and *L. luschani* show the most extreme contractions across all scenarios, while *L. antalyana* shows limited potential for upslope expansion under moderate scenarios. When all variables, including topographic and anthropogenic predictors, are included (Figure 3), the predicted distributions narrow further, particularly in areas with a high human footprint, indicating that anthropogenic pressures compound climate‐driven habitat loss. Variable importance plots (Figure 4) show that precipitation seasonality (bio15), mean temperature of the warmest quarter (bio10), and elevation consistently rank as the strongest predictors across species, confirming the importance of both thermal and hydrological constraints on *Lyciasalamandra* distributions. Species‐level model performance metrics and projected changes in suitable habitat area under all scenarios are summarised in Table 1.

**TABLE 1:** Species‐level model performance and projected changes in suitable habitat area.

Species	AUC	TSS	Current area (km^2^)	SSP3‐7.0 (km^2^)	SSP5‐8.5 (km^2^)	% change SSP5‐8.5
*L. antalyana*	1.000	0.993	15,764.87	64,619.71	70,151.24	+345.1%
*L. antalyana × billae*	1.000	0.998	7667.95	36,724.55	42,434.78	+453.5%
*L. atifi*	0.998	0.970	83,765.14	25,524.33	22,197.35	−73.5%
*L. billae*	0.997	0.969	36,933.73	12,838.38	8370.17	−77.3%
*L. luschani*	1.000	0.998	20,954.00	4691.41	3111.95	−85.1%
*L. fazilae*	0.999	0.993	13,431.19	3036.09	2689.43	−80.0%
*L. flavimembris*	1.000	0.998	11,953.13	3122.11	1707.88	−85.7%
*L. helverseni*	1.000	0.999	1617.44	358.02	56.24	−96.5%

*Note:* Habitat area in km^2^. % change relative to the current suitable area. 2050 = mean 2041–2060; 2070 = mean 2061–2080.

Abbreviations: AUC, Area Under the Curve; SSP, Shared Socio‐economic Pathway; TSS, True Skill Statistic (mean ± SD across 10 bootstrap replicates).

### 3.1 Niche Overlap

The highest niche similarity was observed between *L. antalyana* and *L*. ×*billae*, with consistently high values across all overlap metrics (Table 2), indicating substantial overlap in their predicted niches and strong environmental and distributional similarity. In contrast, *L. helverseni* and *L. luschani* showed the lowest overlap across all metrics (Table 2), indicating minimal niche sharing consistent with their distinct ecological specialisations and geographic separation. Other notable overlaps include *L. atifi* and *L. billae* (moderate similarity) and *L. fazilae* and *L. flavimembris* (high overlap), as detailed in Table 2.

**TABLE 2:** Pairwise ecological niche overlap indices among the *Lyciasalamandra* species. Bold rows indicate the highest and lowest overlap pairs of greatest conservation relevance.

Species 1	Species 2	*D*	*I*_mod_	*I*_cor_	*R*	*O*	BC	COR
***L. antalyana***	***L. antalyana ×billae***	**0.71**	**0.79**	**0.91**	**0.88**	**0.85**	**0.35**	**0.75**
*L. antalyana* × *billae*	*L. atifi*	0.48	0.67	0.78	0.71	0.48	0.24	0.52
*L. atifi*	*L. billae*	0.54	0.69	0.81	0.75	0.58	0.27	0.70
*L. billae*	*L. fazilae*	0.50	0.68	0.79	0.73	0.49	0.25	0.58
*L. fazilae*	*L. flavimembris*	0.68	0.77	0.89	0.86	0.85	0.34	0.56
*L. flavimembris*	*L. helverseni*	0.37	0.57	0.63	0.55	0.16	0.18	0.59
***L. helverseni***	***L. luschani***	**0.30**	**0.53**	**0.57**	**0.48**	**0.12**	**0.15**	**0.15**

*Note:* *D* = Sørensen similarity coefficient (0–1; proportion of shared predicted presence); *I*_mod_ = Index of Model Similarity (agreement between suitability surfaces); *I*_cor_ = Index of Correlation (linear correlation of suitability values); *R* = Range Overlap (proportion of range 1 overlapping range 2); *O* = Overall niche overlap (symmetric range overlap); BC = Bhattacharyya coefficient (probability distribution similarity); COR = Pearson/Spearman correlation of suitability values. All indices scaled 0–1; higher values indicate greater overlap. Bold rows indicate the species pair with the highest overlap ( *L. antalyana* × *L*. × *billae*) and the lowest overlap ( *L. helverseni* × *L. luschani* ), representing the conservation‐priority contrasts discussed in the text.

## 4 Discussion

This study assesses the potential impacts of climate change on the distribution and habitat suitability of Mediterranean endemic salamander species in southwestern Türkiye. The results indicate that climate‐driven environmental change will make suitable habitats less accessible, likely causing both elevational shifts and, in some species, the complete loss of suitable range.

Consistent with recent findings (Yousefkhani et al. 2023), five out of seven *Lyciasalamandra* species are projected to undergo significant range contractions under both moderate and pessimistic emissions scenarios, while only *L. antalyana* may experience limited range expansion as the suitable climate shifts northward and upward.

Our projections must, however, be interpreted in the context of rapidly changing disturbance regimes in Mediterranean forests, particularly wildfires. In historically fire‐adapted *Pinus brutia* systems, past fires were often of moderate intensity and limited extent, contributing to structural heterogeneity and nutrient cycling (Pausas and Keeley 2019; Keeley et al. 2020). Today, hotter and drier conditions, combined with fuel accumulation and human ignitions, are driving larger and more severe fires that can remove entire forest canopies, consume organic soil layers, and disrupt soil‐dependent faunal communities, including salamanders (Turco et al. 2017; Moreira et al. 2020; Certini 2005). Evidence from the 2021 M‐fires in southwestern Türkiye shows that these events can directly destroy microrefugia for *Lyciasalamandra* and markedly alter local habitat quality, even where broad‐scale climatic suitability persists (Gülçin et al. 2022). Niche similarity analyses highlighted striking heterogeneity in ecological overlap within this group. High niche overlap between *L. antalyana* and *L. antalyana* ×*billae* (Table 2) indicates these species share similar environmental requirements, raising the possibility of increased competitive pressure for remnant habitat if ranges contract simultaneously—though direct evidence of competition would require demographic or behavioural data beyond the scope of this study, a pattern mirrored by Rödder et al. (2011). By contrast, *L. helverseni* and *L. luschani* exhibited minimal overlap across all metrics (Table 2), consistent with their distinct ecological specialisations and evolutionary divergence (Veith et al. 2016; Scott et al. 2025). Such variation underscores the need for tailored, species‐specific conservation actions, rather than generalised strategies.

Patterns of relative species richness provide further insight into possible community responses. The observed spike in local richness projected under the extreme SSP5‐8.5 scenario for 2050–2070 does not reflect a true gain in biodiversity but instead results from multiple species being squeezed into shrinking pockets of suitable habitat. This ‘refugia compression’ could temporarily increase co‐occurrence among ecologically similar species, potentially intensifying resource competition, though the actual outcome will depend on species‐specific dispersal capacity, behavioural flexibility, and microhabitat partitioning. Given the limited dispersal capacity of *Lyciasalamandra* (Veith et al. 2016; Scott et al. 2025), actual increases in co‐occurrence are unlikely. Instead, increased co‐occurrence stress and population declines are plausible outcomes unless habitat connectivity and microrefugia are proactively secured.

In fire‐prone landscapes, these microrefugia are increasingly shaped not only by climate and topography but also by forest management decisions taken in the immediate post‐fire phase. Artificial regeneration practices, such as deep mechanical soil preparation, ploughing and large‐scale removal of coarse woody debris and rock piles, can obliterate the very underground cracks, root channels, and shaded surface structures that allow salamanders to estivate underground during summer and survive drought (Wells 2007; Ultsch 1989; Lindenmayer et al. 2000). By contrast, low‐impact natural regeneration, retention of burned logs and rocks, and strict protection of moist microsites (springs, seeps, boulder fields) can sustain suitable microhabitats even in otherwise heavily burned landscapes (Certini 2005; Lindenmayer and Franklin 2002; Pausas and Keeley 2019). Therefore, the same fire event can have dramatically different outcomes for *Lyciasalamandra* depending on whether post‐fire forestry amplifies or mitigates microhabitat degradation.

In practice, this means that post‐fire silviculture in *Pinus brutia* forests within the ranges of *Lyciasalamandra* should prioritise natural regeneration wherever possible, and only employ artificial regeneration under strict constraints on soil disturbance and microhabitat removal (Gezer 1986; Boydak 2004; Lindenmayer et al. 2000). Shallow soil treatments, spot‐planting and hand‐planting during the driest summer months, combined with the retention and, where feasible, deliberate clustering of large rocks, logs and dead wood, can help recreate shaded and moist refuges that support salamander survival and recolonisation (Lindenmayer and Franklin 2002; Pausas and Keeley 2019). Equally important is the establishment of buffer zones (e.g., ≥ 30–50 m) around springs, seeps, and rock outcrops that are likely to function as key microrefugia and stepping stones within broader habitat networks. The concept of ‘climatic debt’ (He et al. 2023) further warns that amphibians' slow adaptive responses may not match the pace of climate change, increasing extinction risk. The documented role of microrefugia in past compensation for climate instability (Veith et al. 2016, 2020; Scott et al. 2025) now underscores their future conservation value, but their function will be diminished without broader landscape connectivity and water security.

Long‐term conservation of *Lyciasalamandra* will require coordinated actions: legal protection and management of microrefugia, strategic restoration of habitat corridors, integration of dynamic climate risk mapping, and ongoing monitoring of population and intervention outcomes in line with the Amphibian Conservation Action Plan (IUCN SSC Amphibian Specialist Group 2024). In parallel, integrating wildfire‐aware management into protected‐area planning and forest policy will be crucial, as climate‐driven changes in fire regimes are likely to interact synergistically with warming and drying, eroding suitable habitat for salamanders (Bowman et al. 2009; Keeley et al. 2012; Moreira et al. 2020). Future research should prioritise the study of physiological tolerance and adaptive capacity to warming, as well as the exploration of novel interventions such as assisted migration, though strong site fidelity and limited dispersal remain barriers (Veith et al. 2016, 2020).

### 4.1 Conservation Implications

Based on our modelling results, we identify five evidence‐based conservation actions for *Lyciasalamandra* in southwestern Türkiye. These recommendations are spatially grounded in the microrefugia map (Figure 5) and the projected habitat trajectories under all SSP scenarios.

*Safeguard microrefugia*: designate key microrefugia as no‐disturbance zones, prioritising sites that remain suitable across all GCM–SSP combinations.

*Wildfire‐aware forestry*: after fires, avoid deep soil preparation and large‐scale removal of coarse woody debris/rock piles; prioritise natural regeneration, retention of logs and boulders, and buffer zones (≥ 30–50 m) around springs/seeps and rock outcrops.

*Fine‐scale connectivity*: maintain short‐distance, cool‐moist “stepping stones” (boulder fields, deep crevices, ravines) between microrefugia; restrict track opening on north‐facing corridors.

*Monitoring*: implement standardised occupancy surveys (late autumn–winter) at microrefugia and use environmental DNA (eDNA) in springs/seeps to detect cryptic persistence.

*Policy*: include *Lyciasalamandra* as a flagship species for moist microhabitats in Mediterranean forest policy, ensuring that post‐fire interventions are evaluated for their impacts on subterranean fauna, not only for erosion control or timber production.

## Author Contributions

**Morteza Naderi:** conceptualization (equal), data curation (equal), formal analysis (equal), funding acquisition (equal), investigation (equal), methodology (equal), project administration (equal), resources (equal), software (equal), supervision (equal), validation (equal), visualization (equal), writing – original draft (equal), writing – review and editing (equal). **Kerim Çiçek:** data curation (equal), formal analysis (equal), investigation (equal), methodology (equal), validation (equal), writing – original draft (equal), writing – review and editing (equal). **Yasin İlemin:** conceptualization (equal), data curation (equal), formal analysis (equal), investigation (equal), methodology (equal), validation (equal), visualization (equal), writing – original draft (equal), writing – review and editing (equal). **Amaël Borzée:** conceptualization (equal), investigation (equal), software (equal), writing – original draft (equal), writing – review and editing (equal). **Hossein Azadi:** formal analysis (equal), investigation (equal), methodology (equal), software (equal), validation (equal), writing – review and editing (equal). **Babak Naimi:** formal analysis (equal), software (equal), validation (equal), visualization (equal), writing – original draft (equal), writing – review and editing (equal).

## Funding

This work was supported by Rainforest Trust under the Rapid Feasibility Award Agreement, 3‐TR‐1312‐24‐0‐r1.

## Conflicts of Interest

The authors declare no conflicts of interest.

## Supporting information


**Table S1:** A complete list of retained and excluded predictors with screening rationale.

## Data Availability

The data underlying the results of this study are available in the Zenodo repository (DOI: https://zenodo.org/records/19712651). In accordance with the journal's exceptions for threatened taxa, spatial coordinates for the salamanders' endangered and legally protected species in Türkiye have been provided at a coarse resolution of 10 km to mitigate the risk of poaching and human persecution. All other biological parameters, environmental covariates, and genetic metrics remain at the resolution used for the analyses to ensure full study replicability.
