# Supplementary material for: Climate Crisis in the Mediterranean Hotspot: Conservation Challenges for Endangered Salamanders in Southern Türkiye
Source: Ecol Evol. 2026 Jul 19;16(7):e73962. doi: 10.1002/ece3.73962 (PMC13381062; doi:10.1002/ece3.73962)
Supplement: Supplementary file 1 — Table S1: A complete list of retained and excluded predictors with screening rationale. [file ECE3-16-e73962-s001.docx]

**Supplementary Table S1:** A complete list of retained and excluded predictors with screening rationale

| **Variable group** | **Candidate variables** | **Screening rule** | **Retained variables** | **Rationale** |
| --- | --- | --- | --- | --- |
| Bioclimatic | 19 WorldClim v2.1 variables (bio1–bio19) | VIF > 10 removed | bio2, bio7, bio8, bio9, bio10, bio13, bio14, bio15, bio18, bio19 (n=10) | Biological relevance to amphibian thermoregulation and water balance; collinearity removed |
| Topographic | Elevation, slope, aspect, TRI | Aspect removed (low ecological relevance for this taxon) | Elevation, slope | Direct influence on microclimate and refuge availability |
| Land cover | Trees, shrubs, grasslands, croplands, urban (Corine 2018) | Urban removed (negligible coverage in study area) | Trees, shrubs, grasslands, croplands | Habitat structure and canopy cover for estivation |
| Anthropogenic | Distance to farmland, forests, roads, villages, water; population density; Human Footprint Index | Correlation r > 0.7 removed | Human Footprint Index | Integrative measure of human pressure; correlated variables removed |
| Resolution | All layers | Resampled to 2.5 arc-min | All at 2.5 arc-min | Consistency across variable groups |
